# The myth of the ‘unaffected’ side after unilateral stroke: Is reorganisation of the non‐infarcted corticospinal system to re-establish balance the price for recovery?

**DOI:** 10.1016/j.expneurol.2012.08.031

**Published:** 2012-12

**Authors:** S. Graziadio, L. Tomasevic, G. Assenza, F. Tecchio, J.A. Eyre

**Affiliations:** a Developmental Neuroscience, Institute of Neuroscience, Newcastle University, Newcastle upon Tyne, UK; b Neurologia Clinica, Università Campus Bio-Medico di Roma, Italy; c LET’S-ISTC-CNR, Dipartimento di Neuroscienze, Ospedale Fatebenefratelli -Isola Tiberina, Rome, Italy; d Dipartmento di Neuroimaging, IRCCS San Raffaele Pisana, Rome, Italy; e AFaR, Dipartimento di Neuroscienze, Ospedale Fatebenefratelli -Isola Tiberina, Roma, Italy

**Keywords:** Motor, Stroke, Corticospinal, Homeostatic plasticity, Recovery, EEG, EMG, Corticomuscular coherence, Symmetry, Reorganisation

## Abstract

**Background:**

Bilateral changes in the hemispheric reorganisation have been observed chronically after unilateral stroke. Our hypotheses were that activity dependent competition between the lesioned and non-lesioned corticospinal systems would result in persisting asymmetry and be associated with poor recovery.

**Methods:**

Eleven subjects (medium 6.5 years after stroke) were compared to 9 age-matched controls. The power spectral density (PSD) of the sensorimotor electroencephalogram (SM1-EEG) and electromyogram (EMG) and corticomuscular coherence (CMC) were studied during rest and isometric contraction of right or left opponens pollicis (OP). Global recovery was assessed using NIH score.

**Findings:**

There was bilateral loss of beta frequency activity in the SM1-EEGs and OP-EMGs in strokes compared to controls. There was no difference between strokes and controls in symmetry indices estimated between the two corticospinal systems for SM1-EEG, OP-EMG and CMC. Performance correlated with preservation of beta frequency power in OP-EMG in both hands. Symmetry indices for the SM1-EEG, OP-EMG and CMC correlated with recovery.

**Interpretation:**

Significant changes occurred at both cortical and spinomuscular levels after stroke but to the same degree and in the same direction in both the lesioned and non-lesioned corticospinal systems. Global recovery correlated with the degree of symmetry between corticospinal systems at all three levels — cortical and spinomuscular levels and their connectivity (CMC), but not with the absolute degree of abnormality. Re‐establishing balance between the corticospinal systems may be important for overall motor function, even if it is achieved at the expense of the non-lesioned system.

## Highlights

► We studied the two corticospinal systems in chronic unilateral strokes and controls. ► We investigated motor cortex (EEG), spine/muscle (EMG) and their coherence (CMC). ► Bilateral symmetrical loss of beta power in EEG and EMG was found in strokes. ► EEG, EMG, CMC symmetry indices were similar to controls and correlated with recovery. ► Balance between the two systems is achieved at the expense of the unlesioned system.

## Introduction

Stroke in the territory of the middle cerebral artery (MCA^2^) often causes serious disturbances within the motor system resulting in acute hemiparesis and often permanent impairment of arm control and dexterity. Extensive studies of cortical function after unilateral MCA stroke indicate that the motor deficits do not arise solely from direct focal damage to the sensorimotor cortex, but that intra and inter-hemispheric reorganisation and abnormal interaction between key areas remote from the infarct also contribute to the pathophysiology (Grefkes and Fink, 2011). The control and coordination of the upper limb involve distributed bilateral subcortical and spinal networks and the pathophysiology of upper limb control after MCA stroke is likely also to involve abnormal reorganisation and interaction within and between these networks. Extensive bilateral reorganisation of the corticospinal system, driven by activity dependent competition between the lesioned and non-lesioned corticospinal systems, has already been described after unilateral perinatal stroke (Eyre et al., 2007; O'Sullivan et al., 1998).

Markers of plasticity normally only expressed in the neonatal brain are re-expressed acutely after adult stroke, involving not only the lesioned but also the non-lesioned cortex (Carmichael, 2003). Functional imaging acutely has shown abnormal excess activation of the motor network in the non-lesioned ipsilateral hemisphere during movement of the hand affected (Rehme et al., 2012). A progressive shift of the hemispheric activation balance from the lesioned to the non-lesioned hemisphere has been demonstrated over the first few months after stroke and the greater degree of shift towards the unaffected hemisphere the less the recovery of function over this time (Calautti et al., 2001). Transcranial magnetic stimulation (TMS) and effective connectivity studies reveal that acutely after stroke the non-lesioned primary sensorimotor cortex exerts increased inhibitory influences on the lesioned sensorimotor cortex (Takeuchi et al., 2005) and functional recovery after unilateral stroke is predicted by the balance between the activity dependent plasticity of each hemisphere (Di Lazzaro et al., 2010). Together these observations are consistent with activity dependent competition between the lesioned and non-lesioned corticospinal systems being reinitiated during recovery from adult onset MCA stroke.

Previous studies demonstrating bilateral plasticity after unilateral MCA stroke have focused on hemispheric reorganisation (Sanes and Donoghue, 2000) and have not yet considered reorganisation of the corticospinal system as a whole. In this study we investigate reorganisation of the lesioned and non-lesioned corticospinal systems in patients who are more than 18 months after unilateral stroke occurring in adult age.

Our hypotheses were that in the chronic phase after adult onset unilateral stroke:

(i) there is bilateral reorganisation of the corticospinal systems, reflected not only at the level of the motor cortex (Sanes and Donoghue, 2000), but also in the spinal motoneurones and motor units.
(ii) there is activity dependent competition between the lesioned and non-lesioned corticospinal systems, indicated by reciprocal reorganisation and persisting asymmetry between the two systems during the chronic phase after stroke.
(iii) the greater the degree of asymmetry between the corticospinal systems the poorer the recovery.

In order to compare the degree of reorganisation at three levels, cortex, motor units and their functional connectivity, both within and between the lesioned and non-lesioned corticospinal systems, we focus on the oscillatory activity of the sensorimotor cortices and of the electromyogram recorded bilaterally over hand muscles and on corticospinal functional connectivity reflected in corticomuscular coherence (CMC) (Graziadio et al., 2010).

## Material and methods

The approval of the Ethical Committee of San Giovanni Calibita Fatebenefratelli Hospital and written, informed consent from all the subjects were obtained.

### Subjects

The stroke group comprised 11 previously right-handed subjects who had suffered a first ever, mono‐hemispheric, MCA territory, ischemic stroke more than 18 months previously, confirmed by magnetic resonance imaging, MRI (3 female, mean age 69 ± 10 years; time elapsed from the stroke, median 6.5 years, range 1.8 to 7.4 years; Table 1). The subjects were selected to have a range of initial deficits (NIH_t0_ 1–16) and degrees of recovery (NIH_t_ 0–6; Table 1). Subjects with peripheral neuropathy, dementia or severe aphasia were excluded.

The control group comprised 9 healthy right‐handed subjects, matched in age with the stroke group (7 female, mean age 68 ± 10 years) with no past history of a neurological disorder.

### Clinical assessments

#### Muscle strength

In the stroke group muscle strength was assessed in wrist flexors and extensors, opponens pollicis (OP), and the first dorsal interosseous of the affected upper limb using the MRC scale and a mean score obtained.

#### Recovery index

Stroke severity was assessed using the National Institute of Health Stroke Scale both acutely (NIH_*t*0_), and at the time of the study (NIH_*t*_). An index of recovery was determined as the fraction of recovery with respect to the total possible (which equals NIH_*t*0_ since NIH_*t*_ in healthy subjects is 0):

$$\mathrm{Recovery}\;\mathrm{index}=\frac{\mathrm{NI}H_{t0}-\mathrm{NI}H_{t}}{\mathrm{NI}H_{t0}}\text{.}$$

### Motor tasks

A simple task involving the corticospinal system was chosen to ensure, as far as possible, that patients and controls could achieve a similar level of motor performance with each hand, to prevent the level of performance becoming a confounder. Subjects sat with their arms supported (flexed at the elbow, forearm semi‐pronated) and opposed their thumb to the other fingers against resistance of a semi-compliant object. The pressure generated was displayed (pressure sensor 40PC100G1A, Honeywell Sensing and Control, Golden Valley, Minneapolis, USA). Subjects first performed a maximum voluntary contraction (MVC) for each hand and then alternating periods of 20 s of steady isometric contraction and rest with each hand. The target level was set to 20% of MVC for each hand, to match between groups and between hands the subjective sense of effort and the level of fatigue. A total of 240 s of contraction and rest were recorded for each hand. 120 s of continuous rest with eyes open was also recorded.

### Neurophysiological recordings

EEG (FP2, FP1, F8, F4, Fz, F3, F7, FC6, FC2, FC1, FC5, T4, C4, Cz, C3, T3, CP6, CP2, CP1, CP5, T6, P4, Pz, P3, T5, PO4, PO3, O2, O1; linked mastoid reference) and surface EMG from the skin over right and left OP muscles were recorded using Ag/AgCl electrodes (band pass filter 0.48–256 Hz, sampling rate 1024 Hz,) and a Micromed System Plus SAM32 (Micromed S.P.A. Mogliano, Veneto, Italy).

### Data analysis

#### Sensorimotor cortex EEG

The bipolar derivations over the contralateral hemispheres showing maximum CMC with the EMG were identified as the cortical component of the corticospinal system for each hemisphere and these bipolar derivations were used for further analysis (Graziadio, et al., 2010).

#### Spinal motoneurons and muscle components

The surface EMG was recorded as a measure of the oscillatory activity of spinal motoneuronal groups and the membrane properties of muscle fibers.

#### Estimation of power spectral density (PSD)

**Fig. 1:**
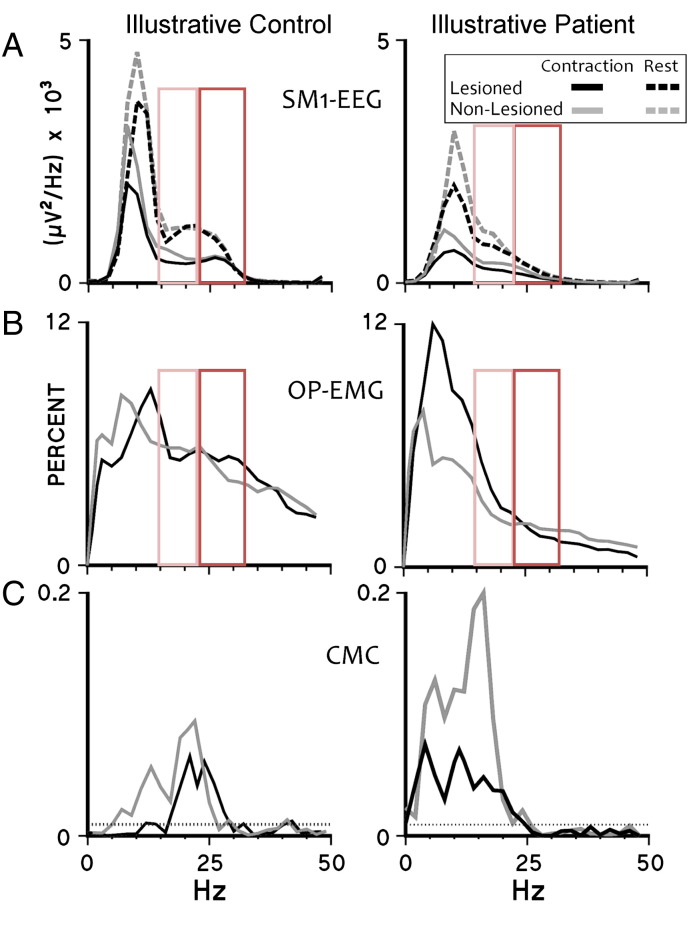
Power spectral density of SM1-EEG, relative OP-EMG and CMC: representative data. Data are plotted from an illustrative control subject (left column) and an illustrative stroke subject (right column). Grey spectra indicate the non-lesioned corticospinal system and their controls, black spectra — the lesioned corticospinal system and their controls. A. PSDs of the contralateral sensorimotor cortex EEG during rest (dashed lines) and during contraction (continuous lines). B. The relative powers of surface EMG recorded over opponens pollicis. C. CMC spectra. The dotted horizontal lines indicate the 95% confidence level for a CMC of zero. Note the bilateral loss of beta activity in this stroke patient compared to the exemplar control subject (enlightened in the red boxes: orange for low beta, red for high beta frequency range; continuous line for significant results on the whole dataset, p < 0.05, dotted for trends, p < 0.1).

Transition periods between rest and contraction were disregarded (0.5 s). 90 s in total of the task for each hand and of rest was analysed. The power spectral densities (PSDs) of EEG (Fig. 1A) and rectified EMG signals (Fig. 1B) were estimated using the Welch procedure (512 data point epochs, Hanning-windowed, 50% overlap). Power was grouped into bands: 2–6 Hz (delta–theta), 8–12 Hz (alpha), 14–22 Hz (low beta), 24–32 Hz (high beta), 34–46 Hz (low gamma) and 54–100 Hz (high gamma) and normalised by the number of frequency points in each band. For the EEG, power in the gamma frequency bands was not analysed, since at rest gamma activity in the EEG is poorly represented and during a steady contraction it is not observed in all subjects (Crone, et al., 1998).

#### Estimation of CMC

CMC was estimated using the method described in Graziadio et al. (2010) (Terry and Griffin, 2008). The number, amplitude and frequency of significant CMC peaks were evaluated (Fig. 1C).

#### Symmetry indices

To investigate the degree of symmetry in oscillatory activity between the lesioned (L) and non-lesioned (nL) corticospinal systems at cortical, spinal/muscular levels we estimated a symmetry index:

$$\mathrm{Sym}I_{x}=1-\sum_{i=2}^{n}\left|\frac{x_{\text{nL}}^{i}-x_{\text{L}}^{i}}{x_{\text{nL}}^{i}+x_{\text{L}}^{i}}\right|$$

(i) for the sensorimotor cortex EEG power at rest (x = sensorimotor cortex EEG PSD) and
(ii) for the relative OP‐EMG power during contraction (x = relative OP-EMG): where *i* is the frequency (*n* = 32 Hz for SM1-EEG and *n* = 100 Hz for OP-EMG).

To estimate the degree of symmetry in cortico-spinal coupling during contraction we computed:

$$\mathrm{Sym}I_{\mathrm{CMC}}=1-\left|\frac{\mathrm{CM}C_{\text{nL}}^{\mathrm{Max}}-\mathrm{CM}C_{\text{L}}^{\mathrm{Max}}}{\mathrm{CM}C_{\text{nL}}^{\mathrm{Max}}+\mathrm{CM}C_{\text{L}}^{\mathrm{Max}}}\right|$$

where $\mathrm{CM}C^{\mathrm{Max}}$ is the amplitude of the highest peak of CMC.

#### Performance of the motor task

We have previously demonstrated a linear relationship between the coefficient of variation of the force and that of the rectified OP-EMG during this task (Graziadio, et al., 2010). Performance was estimated as: $\mathrm{Performance}=1-\frac{\mathrm{std}\left(\mathrm{EM}G_{\text{Rect}}\right)}{\mathrm{mean}\left(\mathrm{EM}G_{\text{Rect}}\right)}\text{.}$

#### Estimation of fatigue during the motor task

The pressure generated, the OP-EMG root mean squared amplitude, and the median spectral frequency of the OP-EMG were used as indictors of fatigue (Jouanin, et al., 2009). Fatigue during each 20 s period of contraction (fatigue within trial) was considered by comparing the mean values during the first 5 s of a contraction with that of the last 5 s; fatigue across the total duration of the study (fatigue along session) was considered by comparing the mean values during first 3 periods of contraction with that of the last 3 periods.

### Statistical analyses

We grouped the non-lesioned corticospinal system of stroke patients (non-lesioned hemisphere and non-paretic hand) and the lesioned corticospinal system of the patients (lesioned hemisphere and paretic hand) irrespective of the side (left or right hemisphere) of the lesion. To control for a potentially confounding effect of left vs. right differences, before approaching the statistical analysis, left and right data were randomly mixed in controls to achieve the same left/right distribution of lesioned/non-lesioned sides in patients. In addition, to prevent possible systematic bias, computation of the PSD was completed blind to whether a hemisphere represented a lesioned or non‐lesioned hemisphere for either group.

The data were normally distributed. Significance was set at p < 0.05, with Bonferroni correction.

Group differences (stroke versus control): a general linear model analysis of variance (ANOVA) with Greenhouse–Geisser correction if required was applied (SPSS 15, SPSS Inc, Chicago, Illinois, USA).

#### Relationship of corticospinal system variables with performance

A univariate ANOVA was used with the corticospinal system variable as a covariate.

#### Relationship of corticospinal system variables with recovery in the stroke group

Spearman correlation was used.

## Results

The clinical data are reported in Table 1. Data are reported as means ± standard errors of the mean.

### Performance of the motor task

There were no differences in performance between control and stroke Groups nor between Tasks (contraction with lesioned or non-lesioned hand) [control: non-lesioned hand, 0.51 ± 0.02; lesioned hand, 0.50 ± 0.03; stroke: non-lesioned hand, 0.50 ± 0.02; lesioned hand, 0.49 ± 0.03], ensuring that differences between lesioned and non-lesioned corticospinal systems and between groups could not be attributed to differences in performance of this simple task.

### Fatigue

There were no significant differences between control and stroke Groups nor Groups ∗ Fatigue interactions (within trial or across trials) in the pressure level [p > 0.26], in the OP-EMG amplitude [p > 0.18] or in the median spectral frequency [p > 0.6], excluding fatigue as a source of differences between Groups or between Tasks (contraction with lesioned or non-lesioned hand).

### Sensorimotor cortex EEG PSD

**Fig. 2:**
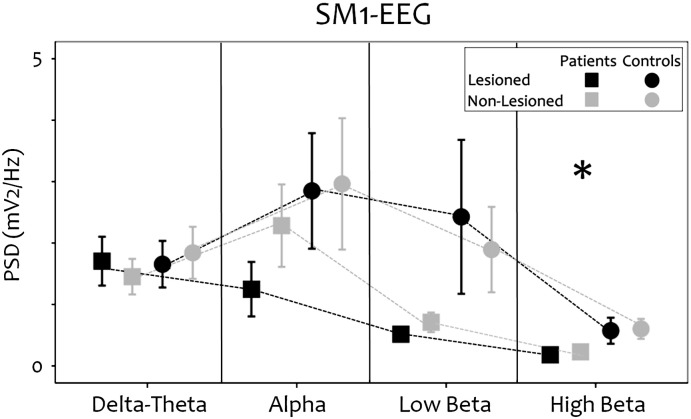
Spectral properties: sensorimotor cortex EEG during rest. Grey lines and symbols represent data from the non-lesioned sensorimotor cortex and black lines and symbols represent data from the lesioned sensorimotor cortex in the stroke group (squares) and the control group (circles). Asterisks indicate significant differences between the groups [p < 0.050].

**Fig. 3:**
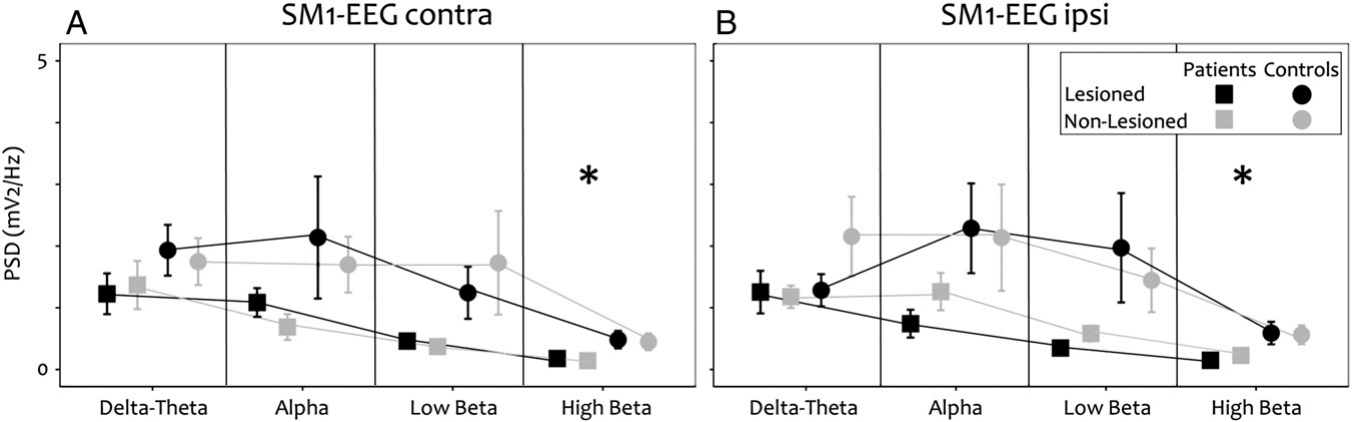
Spectral properties: sensorimotor cortex EEG during unilateral contraction. Grey lines and symbols represent data from the non-lesioned sensorimotor cortex and black lines and symbols data from the lesioned sensorimotor cortex in the stroke group (squares) and the control group (circles). Asterisks indicate significant differences between the groups (p < 0.050). A. SM1-EEG contra — the hemisphere contralateral to the contracting hand B. SM1-EEG ipsi — the hemisphere ispilateral to the contracting hand.

There was decreased power in high beta in the strokes compared to controls [non-lesioned hemisphere: p = 0.025; lesioned hemisphere: p = 0.018] and a trend for decreased power in low beta [non-lesioned hemisphere: p = 0.07; lesioned hemisphere: p = 0.07] at rest and during the motor task (Figs. 2 and 3).

### OP-EMG PSD

#### Total OP-EMG power

During rest there was no significant difference between Groups [p = 0.31] nor a Groups ∗ State (voluntary contracted hand, resting hand) interactions [p = 0.25], indicating that, even if mirroring did occur occasionally, its incidence was similar across stroke and control groups. No difference between Groups nor between Tasks was found during voluntary contraction [p = 0.124, control: 0.35 ± 0.17 mV^2^, stroke: 0.72 ± 0.1 mV^2^].

#### Relative OP-EMG PSD

**Fig. 4:**
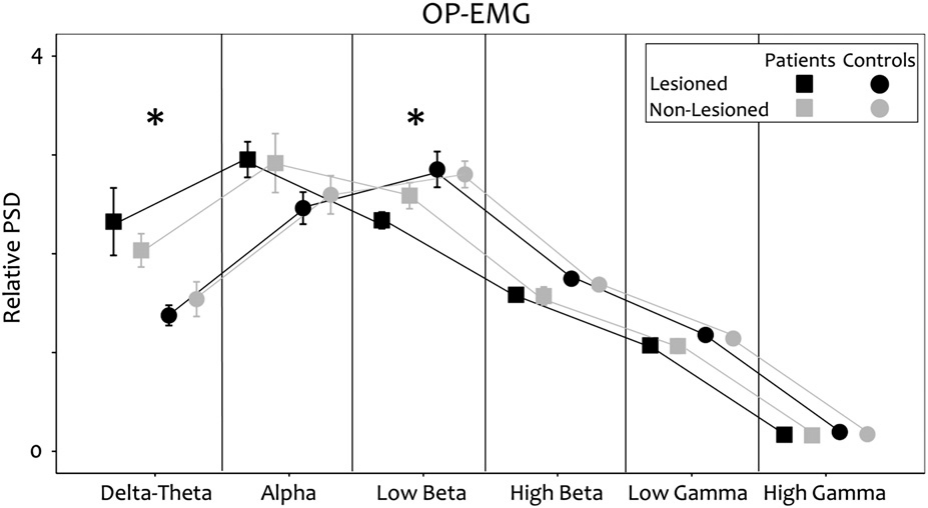
Spectral properties: OP-EMG. During unilateral contraction of the hand muscle contralateral to the non lesioned hemisphere (grey lines and symbols) or to the lesioned hemisphere (black lines an symbols) in the stroke group (squares) and the control group (circle).

In the stroke group there was increased power in 2–7 Hz (delta/theta; p = 0.019) and reduced power in 13–23 Hz (low beta; p = 0.048; Fig. 4) during contraction of the paretic and the non-paretic hands compared to controls.

### Contralateral CMC

No difference was found between stroke and control groups in CMC amplitude [p = 0.645] and peak frequency [p = 0.135]. Stroke subjects had a trend [p = 0.058] towards a greater number of significant peaks in both the lesioned [control: 1.7 ± 0.3, stroke: 2.5 ± 0.3,] and non-lesioned systems [control: 1.5 ± 0.3, stroke: 2.2 ± 0.2].

### Symmetry indices

**Fig. 5:**
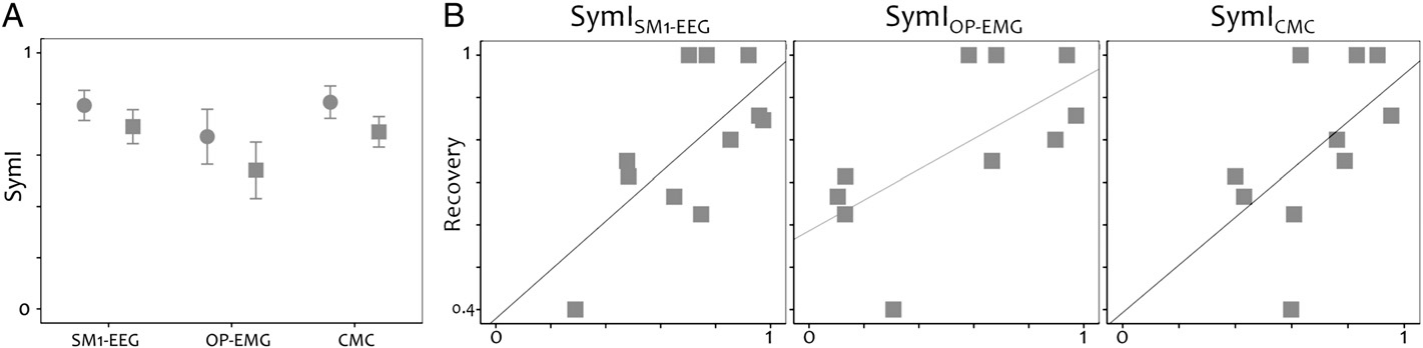
Symmetry indices (SymI). A. Comparison of the symmetry indices in the sensorimotor cortex at rest (SM1-EEG), in the surface EMG recorded over opponens pollicis muscle (OP-EMG) and in the corticomuscular coherence (CMC) between stroke patients (squares) and controls (circles). B. Relationship between recovery and the three symmetry indices.

There were no significant differences between stroke and control groups in the symmetry indices [p = 0.639, Fig. 5A]. SymI_OP-EMG_ correlated with SymI_SM1‐EEG_ [r = 0.555, p = 0.017] and with SymI_CM*C*_ [r = 0.776, p < 0.0011]; SymI_SM1‐EEG_ was correlated with SymI_CMC_ [r = 0.436, p = 0.05, Fig. 3].

### Relationship between performance and corticospinal system variables

**Fig. 6:**
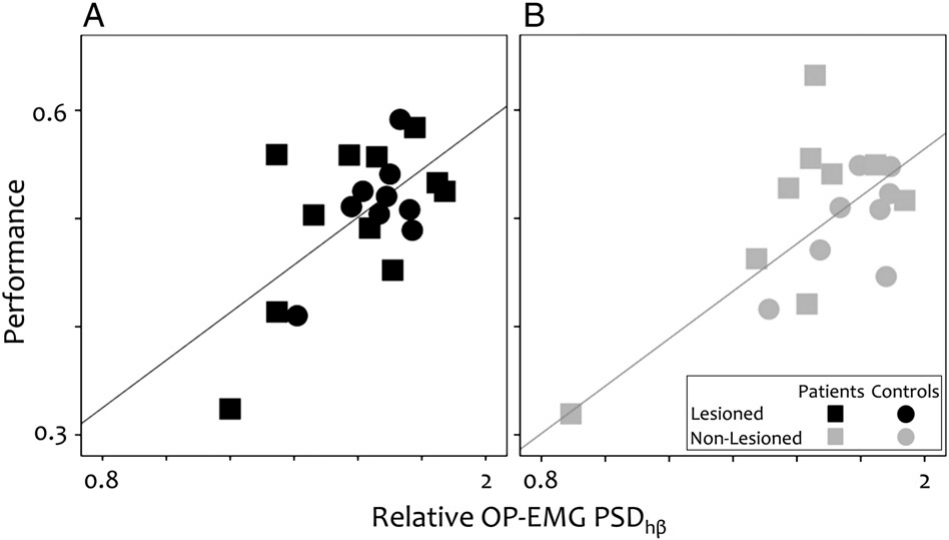
Relationship between motor performance and relative OP-EMG power in high beta. The correlation across both the stroke group (squares) and the control group (circles). A. The non-lesioned system. B The lesioned system.

Relative OP-EMG power in high beta correlated positively with performance in both the lesioned [p = 0.018, B = 0.21, Fig. 6A] and the non-lesioned systems [p = 0.016, B = 0.26, Fig. 6B]. No interaction between OP-EMG power in high beta and Group was observed demonstrating no difference between the stroke patients and controls [p > 0.2].

### Relationship between corticospinal variables and recovery

The recovery index correlated with the SymI_OP-EMG_ [p = 0.023, r = 0.706], SymI_SM1‐EEG_ [p = 0.048, r = 0.606] and SymI_CMC_ [p = 0.023, r = 0.706; Fig. 5B].

## Discussion

### Main findings and conclusions

We began this study with the hypothesis that reorganisation of the corticospinal system after unilateral stroke would be activity driven, leading to marked asymmetry between the more active non‐lesioned system and the less active lesioned system in the chronic phase (Tecchio, et al., 2005; van Putten and Tavy, 2004). Whilst we have demonstrated significant differences between the control and stroke groups at the level of the cortex and also at the spinomuscular level, we report for the first time that the changes occurred to the same degree and in the same direction in the lesioned and non-lesioned corticospinal systems, and also occurred to the same degree between the different levels of the corticospinal system within each subject. Only the degree of symmetry between the corticospinal systems at the three levels (cortex, spinomuscular and their connectivity) correlated with recovery, assessed using the global NIH score, indicating the potential importance of re-establishing balance between the interdependent networks of the two corticospinal systems for overall motor function.

### Bilateral increase of low frequency power of the OP-EMG

In stroke patients the OP-EMG of both hands had increased power in the lower frequencies (2–7 Hz) compared to controls (Fig. 4). The power spectrum of the surface EMG is determined by the number of active motor units within recording range of the electrodes and their frequency content. There is no evidence of a consistent reduction in the firing rate of motor neurons after stroke (Pierrot-Deseilligny and Burke, 2005) to explain a significant shift to lower frequencies. The frequency content of motor unit action potentials is related to the muscle fibre conduction velocity (Rau, et al., 2004). Muscle fibre atrophy from underuse of the affected hand could not explain our findings because similar changes were also observed in the non-paretic hand, where if anything, over use is likely to have occurred. A shift to lower frequencies in the surface EMG would be predicted from the selective loss of type II muscle fibres demonstrated to occur acutely in the paretic hand (Lukács, et al., 2008), since this would lead to larger relative contributions from slow conducting type I motor units. Our observation of a shift to lower frequencies in the EMG of both hands would be consistent with a loss in the chronic phase of type II muscle fibres also in the non-paretic hand, to match that occurring acutely in the paretic hand.

### Bilateral decreased power in beta frequencies in the SM1-EEG and in the OP-EMG

Stroke patients had reduced power in the beta frequencies in both the sensorimotor cortex EEG and in the OP‐EMG and performance of our task with either hand correlated with the degree of preservation of high beta power in the OP-EMG. The beta frequency has particular relevance to the motor system, since it is optimal for information transfer in large‐scale or widely separated networks, such as those in the corticospinal system (Kopell, et al., 2000). Furthermore, beta frequency resonance occurs in the key neural cortical and subcortical networks for movement control and its power is modulated before and during motor tasks (Murthy and Fetz, 1996; Sanes and Donoghue, 1993; Schalow, 1993; Soteropoulos and Baker, 2006). Bidirectional information flow in corticomuscular coherence occurs in the beta frequency (Meng, et al., 2008) and provides support for the proposal that coherent networks oscillating in the beta frequencies form interconnected loops from the cortex to the periphery and back (Graziadio, et al., 2010; Witham, et al., 2011). In our study there is evidence for detuning (Graziadio, et al., 2010) of information transfer in the corticospinal network after stroke, with a trend towards a greater number of significant CMC peaks in the stroke group during isometric contraction for both the lesioned and non-lesioned system.

### Bilateral corticospinal system reorganisation and its relationship to recovery

Functional MRI studies have previously demonstrated that rebalancing activity between the infarcted and non-infarcted hemispheres in the chronic phase is associated with better recovery after stroke (Calautti, et al., 2010). We are, however, the first to demonstrate that this concept extends also to the corticospinal systems, where in the chronic phase after stroke, we have demonstrated re-establishment of the balance in activity both within and between the corticospinal systems, not only at the cortex but also at spinomuscular level, and that this is achieved, at least in part, at the expense of reorganisation of the non-lesioned system.

**Fig. 7:**
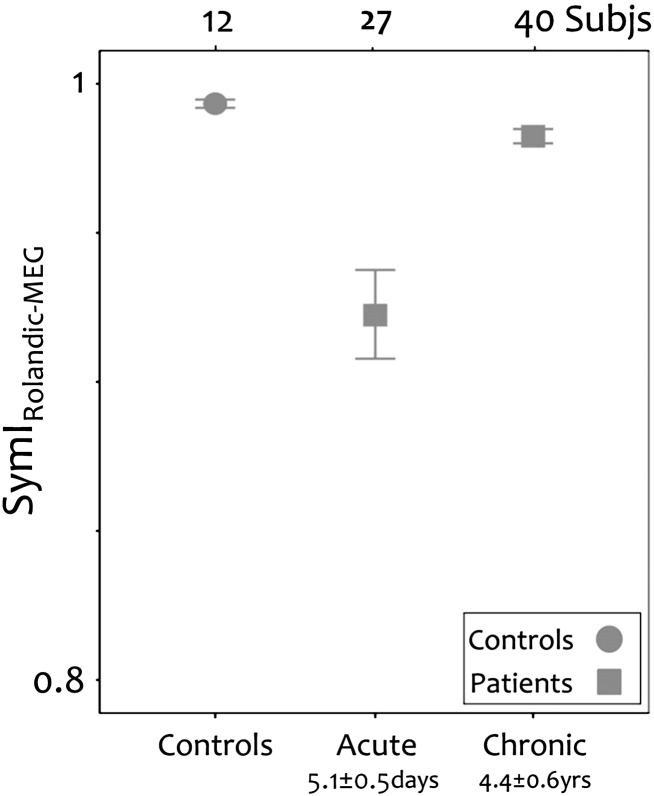
Symmetry indexes (SymI_Rolandic-MEG_) for additional MEG datasets from Tecchio et al., 2005, 2006. Comparison of the symmetry indices for the rolandic area at rest between controls and between stroke patients during acute (Tecchio, et al., 2005) and chronic phases (Tecchio, et al., 2006) of recovery.

Previous studies have demonstrated marked asymmetry acutely in EEG/MEG power between the lesioned and non-lesioned hemispheres (Tecchio, et al., 2005; van Putten and Tavy, 2004). One of the authors of our current study had previously reported reduced power in the beta frequencies during rest in MEG recorded over the rolandic area in both the acute and chronic phases after stroke (Tecchio, et al., 2005, 2006). In the first study of 32 patients within one week of stroke, beta power in the lesioned hemisphere was significantly lower than in the non lesioned hemisphere (Tecchio, et al., 2005) but in a study of 56 patients who were more than one year after stroke, paired comparison within patients showed no differences in beta power between hemispheres (Tecchio, et al., 2006). We calculated a SymI for the MEG power for subjects from both these studies (Tecchio, et al., 2005, 2006) to determine if there was a significant change from the acute period to the chronic period in the symmetry of rolandic area MEG power after stroke. The patients in the chronic phase (Tecchio, et al., 2006) had significantly higher SymIs than those in the acute phase (Tecchio, et al., 2005) [Kolmogorov–Smirnov exact test, p < 0.001; Fig. 7]. We considered whether this change could simply occur because those studied chronically (Tecchio, et al., 2006) had less impairment overall than those studied in the acute phase (Tecchio, et al., 2005); however this is unlikely since the patients studied in the chronic phase had significantly greater impairment acutely [p < 0.0001] than those studied in the acute phase, as measured by NIH scores assessed in both subject groups in the period immediately after the stroke.

In the present study the only variables correlating with recovery were the symmetry indexes. When reviewing the data of Tecchio et al. (2006) we also found in those subjects a positive relationship between the SymI, estimated from rolandic area MEG power and the recovery index [one-tailed Spearman correlation, p = 0.007, r = 0.385].

Complex behaviors emerge through the integration of neural circuits usually comprising bilateral components of the peripheral and central nervous systems. Such neural circuits must maintain stable function in the face of many plastic challenges during learning, development and aging. Recent work has shown that destabilizing influences are counterbalanced by homeostatic mechanisms that act to stabilise neuronal and circuit activities (Turrigiano, 2012). Our findings imply that homeostatic plasticity may be induced during recovery from focal, unilateral stroke to re-establish balance both within and between the interdependent lesioned and non-lesioned corticospinal systems. Since the degree of global recovery in the chronic phase correlated with the degree symmetry achieved and not with the absolute level of abnormality of the corticospinal variables, this implies that imbalance between the corticospinal systems contributes to the pathophysiology of movement disorders after stroke. This proposal is supported by previous observations in a longitudinal study of middle cerebral artery stroke in neonates where the degree of abnormality in the infarcted corticospinal system acutely was not predictive of outcome but rather the greater the asymmetry between the corticospinal systems at two years, the worse the motor outcome (Eyre, et al., 2007).

### Could reorganisation of the non-lesioned corticospinal system contribute to ipsilesional upper limb dysfunction?

More than 30 years ago the Norwegian neuroanatomist Brodal observed that his right sided handwriting was impaired after he suffered a right hemisphere stroke (Brodal, 1973). Numerous studies have since demonstrated abnormal ipsilesional upper limb function when performing skilled motor tasks, irrespective of whether the lesion is cortical or subcortical, and currently there is no consensus on what types of deficit lead to impairment of ipsilateral hand function, independent of the site of the lesion (Nowak, et al., 2007; Winstein and Pohl, 1995). Our findings raise the hypothesis that homeostatic plasticity induced during recovery to maintain stability within and between corticospinal systems does so, at least in part, at the expense of fine motor function in the ipsilesional arm and hand, by driving reorganisation of the non-lesioned corticospinal system to mirror or balance that of the lesioned system. If our hypothesis is substantiated in longitudinal studies, it would have significant implications since rehabilitation paradigms would need to consider not only benefit to the paretic arm, but also potential benefit or harm induced in the ipsilesional arm and hand.

## Conflict of interest

Graziadio S, Tecchio F, Assenza G, Tomasevic L, Eyre JA report no disclosure.

## Authorship definition

Graziadio S contributed to study design, data analysis and drafting of the manuscript.

Tomasevic L contributed to data analysis and revising of the manuscript.

Assenza G contributed to revising of the manuscript.

Tecchio F contributed to study design, data analysis and drafting of the manuscript.

Eyre JA contributed to study design, data analysis and drafting of the manuscript.

## Role of the funding source

The funders of the study had no role in the study design, data collection, analysis and interpretation, writing the paper or in the decision of where to publish the paper.

## Figures and Tables

**Table 1:** Stroke group: clinical information. F indicates female, M male.

Age (yrs)	Sex	Lesioned hemisphere	Time from stroke (yrs)	NIH_t_	NIH_t0_	MRC	Lesion site
59	F	Left	7.1	6	16	3.5	Subcortical
59	M	Left	7	1	7	5	Cortical
72	M	Left	6.7	0	4	5	Subcortical
83	M	Left	6.5	0	11	5	Cortical–subcortical
69	F	Left	3.1	2	13	5	Cortical–subcortical
57	F	Right	7.4	4	7	4.2	Cortical–subcortical
74	M	Right	7.2	0	1	5	subcortical
72	F	Right	6.3	4	14	3	Cortical–subcortical
69	M	Right	6.1	1	3	5	Cortical
60	M	Right	1.9	3	12	4.5	Cortical–subcortical
82	M	Right	1.8	1	5	4.7	Cortical–subcortical

## Footnotes

2 CMC = corticomuscular coherence; OP = opponens pollicis; MVC = maximum voluntary contraction; PSD = power spectral density; L = lesioned; nL = non-Lesioned; SymI = symmetry indexes; SM1 = sensorimotor cortex.
